# Intermixed Time-Dependent Self-Focusing and Defocusing Nonlinearities in Polymer Solutions

**DOI:** 10.1021/acsphotonics.1c01917

**Published:** 2022-02-01

**Authors:** Athanasios Bogris, Nikolaos A. Burger, Konstantinos G. Makris, Benoit Loppinet, George Fytas

**Affiliations:** † FORTH, Institute of Electronic Structure and Laser, 70013 Heraklion, Crete, Greece; ‡ Department of Materials Science and Technology, University of Crete, 70013 Heraklion, Crete, Greece; § Department of Physics, University of Crete, Heraklion 71003, Greece; ∥ Max-Planck Institute for Polymer Research, 55128 Mainz, Germany

**Keywords:** nonlinear optics, soft matter, photoreactive polymer solutions, self-focusing, self-defocusing

## Abstract

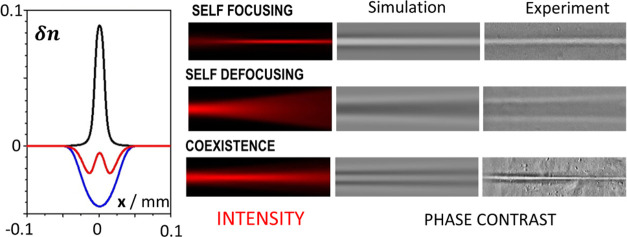

Low-power visible light can lead to spectacular nonlinear effects in soft-matter systems. The propagation of visible light through transparent solutions of certain polymers can experience either self-focusing or defocusing nonlinearity, depending on the solvent. We show how the self-focusing and defocusing responses can be captured by a nonlinear propagation model using local spatial and time-integrating responses. We realize a remarkable pattern formation in ternary solutions and model it assuming a linear combination of the self-focusing and defocusing nonlinearities in the constituent solvents. This versatile response of solutions to light irradiation may introduce a new approach for self-written waveguides and patterns.

## Introduction

When light impinges a nonlinear material with refractive index n, the feedback on its propagation will exert self-focusing (defocusing) in the case of a local increase (decrease) in refractive index, δ*n*. The specific light–matter interactions define the mechanism through which photon influx modifies the materials’ refractive index. Different physical mechanisms lead to nonlinearity including modification of electronic configurations and molecular orientation in dielectric materials;^1,2^ photothermal effects with local temperature modulation of the refractive index, d*n*/d*T*; mass transport (thermophoresis); or photochemical processes triggering chemical modifications (cis–trans conformation transitions, photobleaching, reactions).^3−7^ The various mechanisms relate to different spatiotemporal scales and amplitudes of the refractive index modulation. The standard Kerr effect results in a relatively low refractive index increase, and nonlinear light propagation requires a high input power. The core of nonlinear optical materials is crystalline solids or simple liquids and requires powerful laser beam actuation. Low-power nonlinear optics demands higher nonlinear coefficients. More complex materials like solutions or dispersions have been considered offering additional channels to trigger nonlinear responses. Liquid crystals^8−12^ and thermal media^13−19^ are arguably the most studied physical systems presenting a high nonlinear response. Thermophoresis, dielectrophoresis, and photochemical response are among the mechanisms investigated (besides molecular orientation in liquid crystals). The large number of degrees of freedom present in more complex materials may also lead to multiplicity of coexisting nonlinearity channels. Carefully crafted systems can therefore be expected to possess a complex nonlinear response not attainable with simpler materials.

The complexity is likely to lead to some nonlocality of the response in space and time, especially through coupling of transport coefficients. In particular, spatial nonlocality is often present in self-focusing. The consequences of the nonlocal responses have attracted large attention during the last decade^8−22^ in an effort to associate nonlinear optics and complex photonics. The spatial nonlocal response allows for long-range effects between coherent nonlinear structures, formation of vortex ring solitons, and novel types of modulational instabilities.^13,14,17^ Because of the broad range of time scale of the temporal response, complex nonlinear media may typically possess a slow response. Moreover, many processes can be time irreversible, and out-of-equilibrium structures are easily formed. Therefore, nonlocality in time response must be considered, especially in the case of irreversible photochemical processes.

Although self-focusing has often been reported, the reverse effect of defocusing has been less studied, and theoretical and experimental^20−22^ studies are mostly in the context of nonlinearity management, pulse and beam shaping. Defocusing is typically triggered by thermal effects as in most materials d*n*/d*T* is negative. In mixtures, thermophoresis can also lead to defocusing depending on the direction of the temperature-gradient-induced transport. A large local decrease in refractive index has been reported in soft-matter systems through thermal effects.^23^

Polydiene solutions have recently emerged as an unanticipated class of photoreactive polymers. They present unexpected strong responses to weak laser irradiation in the visible region.^24−26^ The transparent polymer solutions have shown a remarkable effect of self-focusing and self-propagating/self-written waveguides. Very strikingly, changing the solvent, for the same polymer solute, can switch the self-focusing to a defocusing response, in spite of the higher refractive index of the polymer.^27^ The physicochemical origin of the observed responses remains to be fully identified. The mechanism is likely to be the result of a combination of several steps, and a model could be complex to establish. But the optical response can be captured and understood at a coarse-grained level in a simple way, as the present article will further show. In particular, the presence of both responses in very similar materials provides the base for the “formulation” of materials with coexisting self-focusing and defocusing.

In this paper, we further investigate this novel type of highly nonlinear medium and highlight two distinct physical characteristics of the responses: nonlocality in time (integrating irreversible refractive index change) and coexistence of self-focusing and defocusing nonlinearities in the same medium occurring at different time scales (time-dependent nonlinear coefficient). The difference in time response allows the elaboration of a complex self-written pattern and modulation of beams. The experimental observations are well reproduced by a simple model considering two independent mechanisms for self-focusing and defocusing.

## Results and Discussion

### Defocusing Response

**Figure 1:**
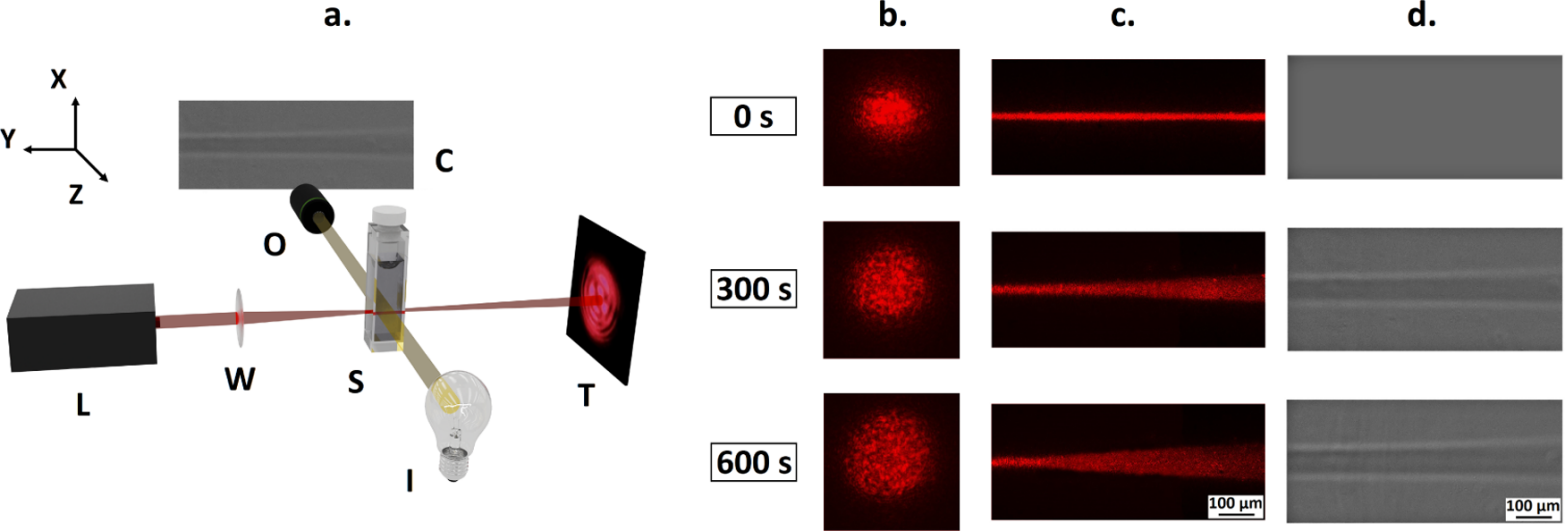
Patterning transparent polymer solutions. (a) Schematic illustration of the experimental setup used for the irradiation of the polymer samples (S) and observation of the induced changes. L is the laser source (671 nm), W is the writing lens, I is the microscope’s illumination, O is the microscope’s objective, C denotes the CCD imaging camera, and T is the spot of the transmitted incident beam through the solution. (b) Time evolution of the transmitted beam spot imaged on a screen. (c) the scattering laser beam at an angle of 90°, and (d) the pattern formation using a blue filter to block the scattered laser light for polybutadiene (PB-390k) 20 wt % solution in tetrahydrofuran (THF). Laser power: 60 mW. Laser propagation axis: left to right.

Solutions of polybutadiene (PB-390) with molecular weight 390 kDa and 20 wt % in THF were irradiated with a laser beam at 671 nm and power up to 110 mW. The laser light was focused at the entrance of the 2 mm cuvette by a 4x lens (focal length *f* = 35 mm), as schematically shown in Figure 1a. The transmitted beam, imaged on a far-field screen, opens up with time and quickly assumes a speckled structure (Figure 1b) before it reaches a steady state after about 600 s. A side view (90°) of the scattered light shows the initially well-collimated beam to open into a divergent beam (Figure 1c) clearly apparent after 300 s. Phase contrast images reveal the formation of a conical stripe-like pattern with refractive index lower than the surrounding nonilluminated solution as indicated by the darker region in the interior of the patterns (Figure 1d). The solution responds to the light irradiation by a local decrease of refractive index (δ*n* < 0) and acts as a diverging lens. The patterns do not fade away when the light illumination is stopped, signaling an irreversible change in the material.

**Figure 2:**
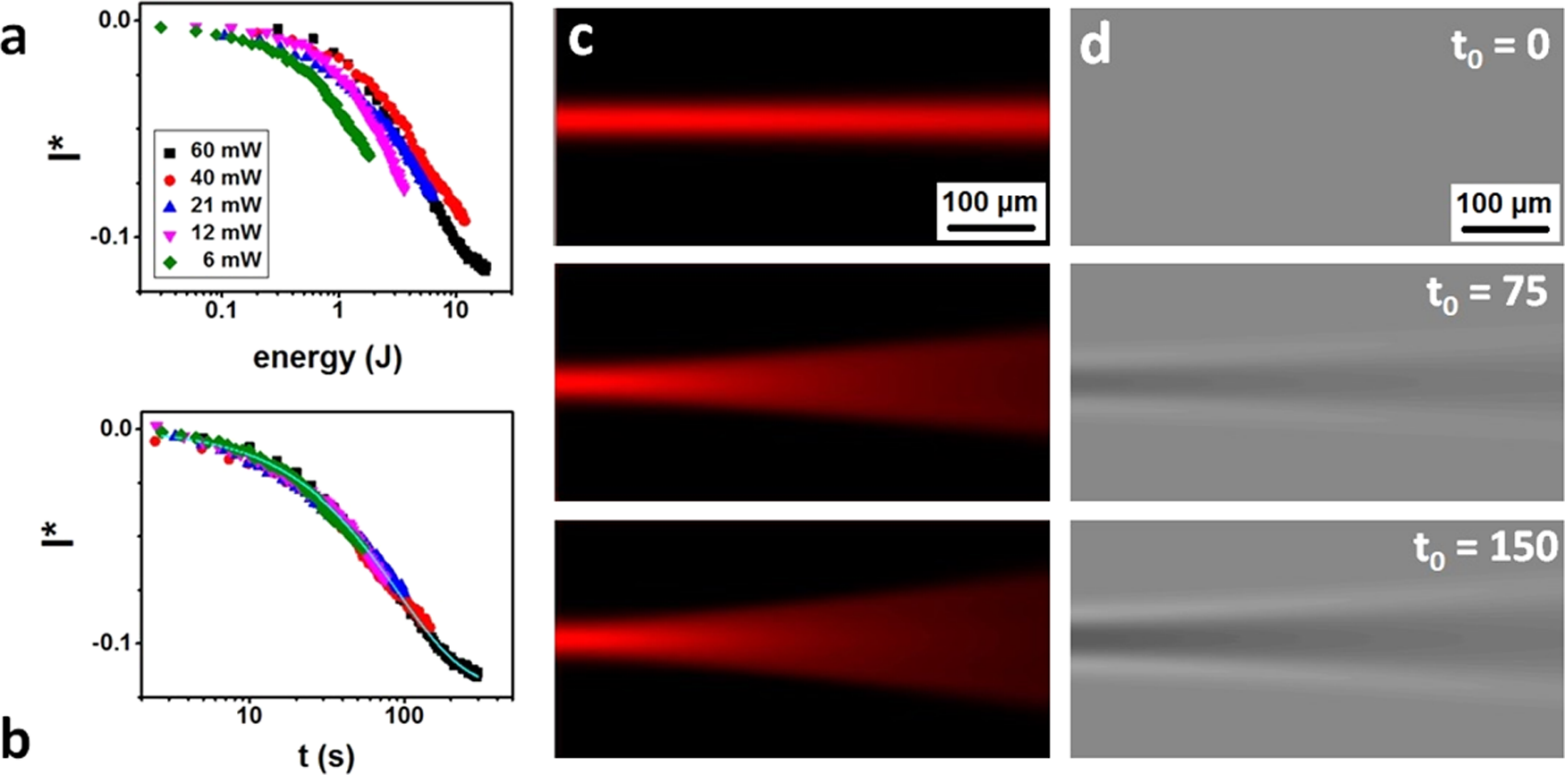
Characterization of the written patterns. Averaged normalized image intensity (I*) for a 20 wt % PB (390k) solution in THF, illuminated by different laser powers at 671 nm plotted as a function of either laser illumination energy influx (a) or rescaled time (b), where time for each curve has been multiplied by a power-dependent factor taken as 1 for the highest power; the shift factor is presented vs power in Figure S2 in the Supporting Information. Colored symbols indicate the different laser powers given in the inset in (a). The solid line in (b) represents the first-order kinetics expression, . Simulation results of the beam’s time evolution (c) and phase images (d) based on the theoretical model. Laser propagation axis: left to right.

The kinetics of the response is characterized by phase contrast microscopy [see the Supporting Information (SI) for details]. As a simple measure of the refractive index variation, we used the normalized image contrast, I*, as a function of the illumination time at different light powers. The time evolution of I* and the refractive index depend on the light power. In fact, a reasonable overlap of the different kinetics is obtained when I* is considered as a function of the laser dose energy (Figure 2a). This superposition implies that the refractive index variation depends on the total number of photons through the irradiated area, denoting the time-integrating character of the response. The overall temporal evolution of I* is well captured (line in Figure 2b) by first-order kinetics , where *I*_∞_^*^ is the intensity contrast at saturation infinite time, *P* is the laser power, *E*_0_ is the energy influx, and β is a small correction factor. Note that I* is defined as the maximum contrast in the phase contrast images and is negative as it is darker than the background. The first-order kinetics with rate being proportional to the laser power is similar to the kinetic model proposed for photoreactive materials^28−30^ with a nonlocality in time and local in space optical nonlinearity. It is also referred to as time-integrating nonlinearity, as the local change of refractive index is proportional to the total number of photons impinging the region of interest. However, instead of an increase, a local decrease of the refractive index with irradiation dose is observed (Figure 2a).

### Modeling

The effect of defocusing nonlinearity on the beam propagation can be described by a scalar nonlinear wave equation. For one-dimensional (more precise 1 + 1 dimensional) problem, the nonlinear wave evolution of a linearly polarized paraxial beam is governed by the paraxial equation of diffraction (Schrödinger type of the parabolic wave equation)
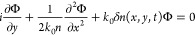
1where Φ is the envelope of the slowly varying optical beam, *x* is the transverse coordinate, *y* is the propagation direction, *k*_0_ is the wavenumber, *n* is the background refractive index, and δ*n*(*x,y,t*) is the refractive index modulation (equivalent to the effective optical potential) that depends on time *t*. Equation 1 is the main paraxial equation that describes the wave’s evolution in a nonabsorbing nonlinear material. For a simple local nonlinearity, δ*n* depends on the intensity |Φ|^2^ with the Kerr nonlinearity term being |Φ|^2^Φ. Hence, the specifics of the material nonlinearity are in the δ*n* term and its dependence on Φ. If the material exhibits nonlocal nonlinearity, like in the present case, δ*n*(*t*) is more complicated and is given by an integral of the optical intensity *I*(*t*) in time, as in eq 2.^28^

For the case of defocusing nonlinearity alone, the evolution of δ*n*_(*x,y,t*) was empirically taken as
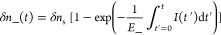
2where δ*n*_s_ is the saturated index difference, *E*_ is the irradiation dose parameter, and *I*(*t*) is the intensity at irradiation time *t* at a given point. Equation 2 represents a time-integrating nonlinearity, where the change in refractive index at a given position is proportional to the total irradiation at this point.^28^Equation 1 was numerically solved at different time steps using a time-dependent split-step Fourier method,^31,32^ providing an intensity *I* = |Φ|^2^ that was in turn used to calculate the new value of δ*n*. The simulation results provide a time evolution of the intensity and refractive index maps. Comparison with experimental data was done on phase contrast images and contrast. Refractive index maps were used to compute a phase contrast image (Figure 2d) and I*.

The simulated evolution of the optical field intensity and the phase image are shown in Figure 2c,d, respectively. The beam opening clearly shows self-defocusing (Figure 2c), and the irradiation leads to the formation of conical patterns of reduced refractive index. The agreement with the experiments is satisfactory, as both the phase image and the intensity distribution from simulations (Figure 2c,d) are qualitatively similar to the experimental evolutions (Figure 1c,d). The beam propagation shows similar opening in both simulation (Figure 2c) and experiment (Figure 1c). The experimentally observed conical pattern (Figure 1d), with a slightly increasing diameter, is also retrieved by the simulations (Figure 2d).

The phenomenological model and the experiment suggest a local decrease of the refractive index upon irradiation that could be attributed to several mechanisms. A decrease of the polymer concentration would be an option, but it seems unlikely in view of the formation of a permanent irreversible material pattern (as explained in SI Section 5). The latter, consequence of polymer cross-linking, would inevitably lead to a polymer concentration increase.^33^ Chemical changes of the polymer, albeit detectable by spectroscopy, are not large enough to lead to a substantial decrease of the solution refractive index. Instead, the local refractive index decrease is likely to result from a local decrease of the solution density caused by the formation of small pockets of gas. Cavitation, as a consequence of a large local pressure gradient, is known to lead to a decrease of refractive index in solids.^34^ In the present case of low power and only marginal absorption, photochemical processes leading to the formation of gas could be at the origin of a density decrease in the concentrated polymer solutions. In such cases, nanobubbles can be trapped in the viscous solution, thereby leading to a decrease in its refractive index. In fact, the observation of gas bubbles in more diluted THF solutions as shown in Figures S3 and S4 can support the decrease of the refractive index. To substantiate this “bubble hypothesis” and complement Figure S4, we visualized eventual small bubbles through their scattering and their evolution during the irradiation. Figure S5 supports the plausibility of our explanation and the generality of the response.

### Coexisting Defocusing and Self-Focusing Nonlinearities

**Figure 3:**
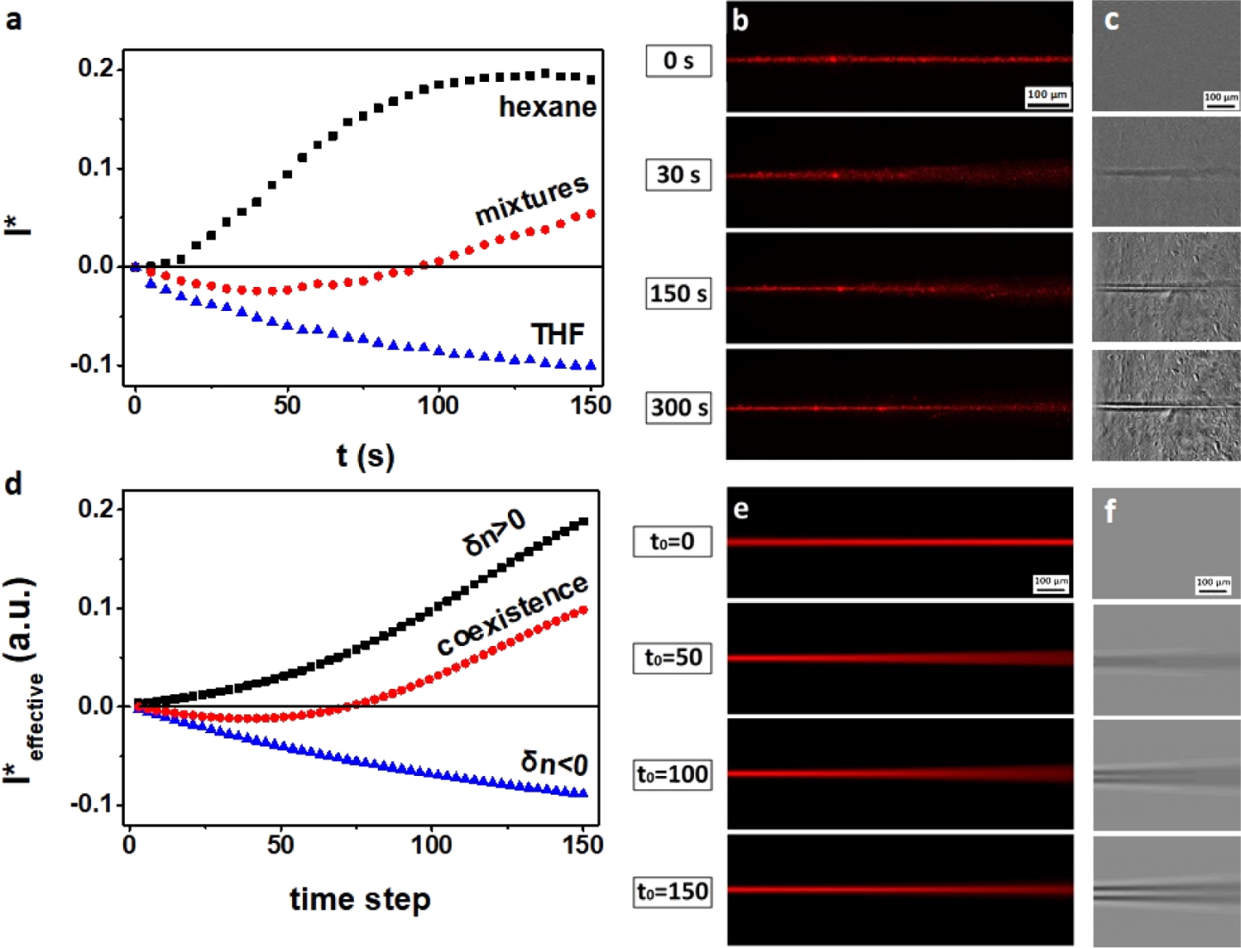
Tuning the written patterns. Normalized image intensity (I*) for PB (390k) 20 wt % solutions in THF, hexane, and THF/hexane mixture as a function of laser illumination time (a) recorded experimentally and (d) simulated numerically at three different THF/*n*-hexane compositions (self-focusing: black, self-defocusing: blue, and transition from self-defocusing to self-focusing: red). (b) Time evolution of the scattering laser beam at 90° and (c) patterning formation in PB (390k) 20 wt % solution in THF (70%)/hexane (30%) binary solvent mixture, irradiated at 671 nm and 60 mW. (d–f) Intensity image and phase image obtained from the numerical simulation simulated analogues of (a–c). Laser propagation axis: left to right.

The presence of the two independent processes and a crossover between them was proven in the case of a binary solvent mixture. Figure 3a illustrates the realization of the two light–matter interactions in the pure solvents (THF and *n*-hexane) and in the binary solvent mixture of 70% THF and 30% hexane (blue triangles) irradiated at 632 nm and 60 mW. THF and *n*-hexane are both good solvents for polydienes and have similar refractive indices. The red curve in Figure 3a represents the phase image contrast I* as a function of time at the entrance of the beam. For the solvent mixtures, a striking change of the slope from initially negative, as in pure THF (Figure 3a, blue curve), to positive, as in *n*-hexane (Figure 3a, black curve), occurred in the solvent mixture rich in THF (70%). The corresponding scattered light patterns (Figure 3b) clearly revealed an early opening followed by the increase of the central part that is typical of the self-focusing by self-written patterns.^25,35,36^ Consistently, the phase contrast image of Figure 3c resembled the THF-like (negative) pattern at early times and transformed to the *n*-hexane-like (positive) pattern growing in the middle of the negative at later times as the irradiation proceeded.

The cosolvency effect on the particular light–matter interactions (Figure 3) suggests the applicability of a coexistence model assuming simple addition of the two, positive and negative, processes. In this ideal mixing proposition, both mechanisms happen independently and simultaneously. For the negative case, we used the integrating nonlinearity model described above. For the positive case, we adopted a model of exponential growth followed by a saturation as observed in the experiments^36^
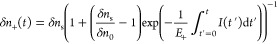
3where *E*_+_ is the dose parameter that controls the early exponential growth rate, δ*n*_s_ is the maximum increase of the refractive index at saturation, and δ*n*_0_ is the minimum refractive index change at time *t* = 0. It corresponds to the initial value that is amplified by the mechanism and empirically found . The model reproduces well typical kinetics reported in previous works in typical polydiene/hexane solutions with, in particular, proper power dependence.^36^ For such time-dependent refractive index modulation, the nonlocal nonlinear wave equation reproduces self-focusing and self-written waveguides.

We simply combine the two nonlinearities through a mixing law: δ*n* = *a*_+_δ*n*_+_ + *a*_δ*n*_, with *a*_+_ and *a*_ being weighting amplitudes. The refractive index at the entrance of the beam is the sum of the two contributions, and the proper choice of parameters provides the observed U-shaped curve as shown in Figure 3e,f. At early times, the faster defocusing dominates, whereas the self-focusing becomes dominant at a later time and further away from the entrance. The pattern shows first the growth of the self-defocused pattern with a self-focused written waveguide appearing in the middle. Away from the beam entrance, the self-written waveguide leads to self-propagation at larger distances, where the self-defocusing has diminished. The pattern and its time evolution compare reasonably well to the experimental observation in mixtures where the linear negative pattern emerges first, but a positive response is soon growing and eventually “collect the light” through self-focusing. The model of sum of two integrating nonlinearities, with different signs and different kinetics, provides a good phenomenological description of the observed patterning in the solvent mixture, bringing an extra proof of the existence of two independent time-integrating nonlinearities in these solutions.

## Conclusions

**Figure 4:**
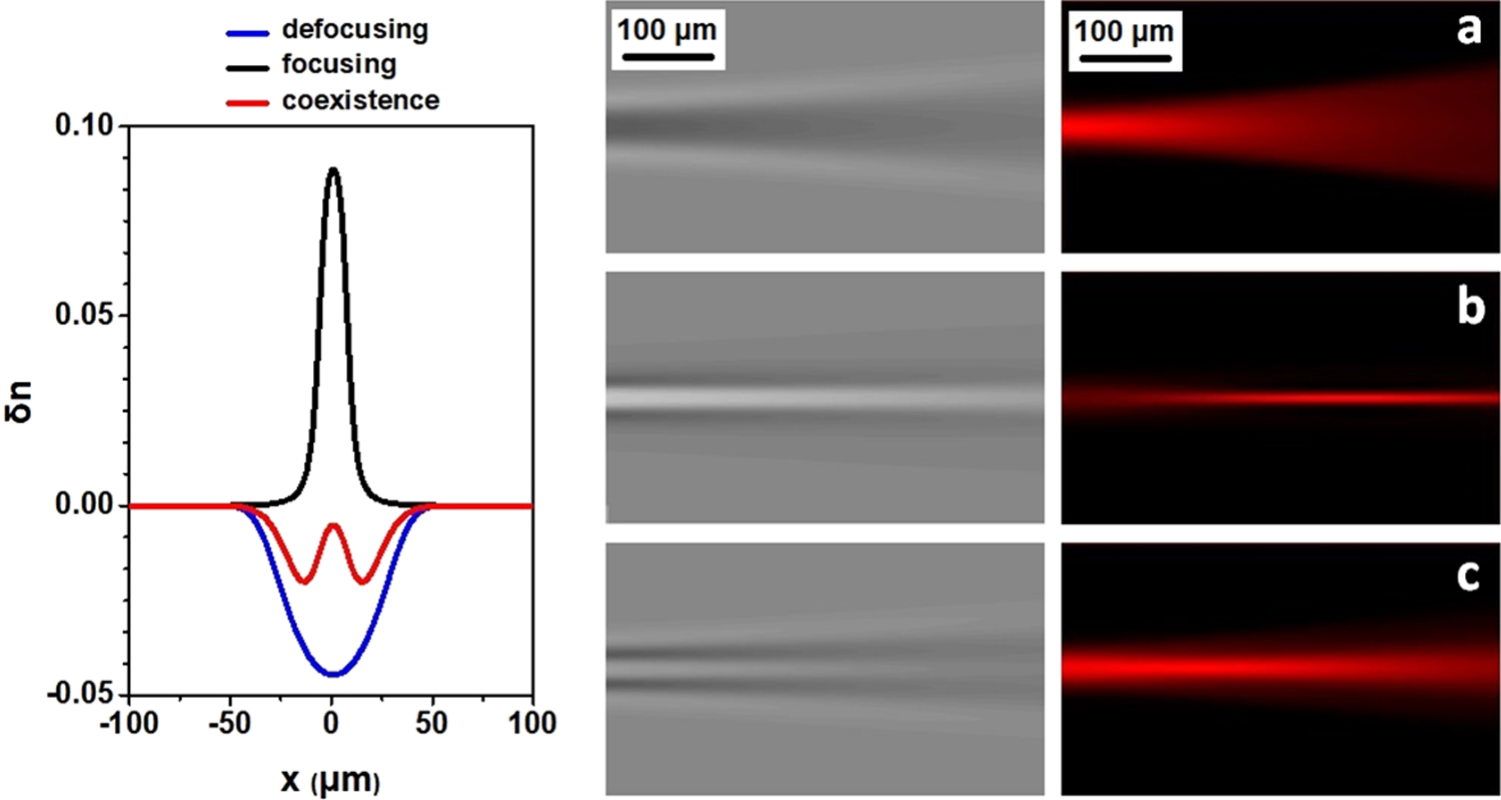
Phase images (left column) and propagation beam (right column) obtained from numerical simulations (*t*_0_ = 150) for (a) self-defocusing case (negative case), (b) self-focusing case (positive case), and (c) coexistence of two cases (solvent mixtures). Corresponding refractive index profiles at *y* = 0 (left graph). Laser propagation axis: left to right.

We demonstrated the presence of two nonlinearities of opposite effects in the same photoreactive polybutadiene solutions. Depending on the solvent used for dispersion, they present either self-focusing or defocusing nonlinearity. Both responses are local in space and nonlocal in time with time-integrating responses, but with different kinetics. The specific light propagation and formation of unique refractive index patterns in mixtures are shown in Figure 4. These are well reproduced by the model of coexistence of self-focusing and defocusing optical responses. The good agreement accredits the hypothesis that the two nonlinearities have different physicochemical origins and can coexist when a mixture of solvents is used. The presented results further highlight the versatility of the optical response of polydiene solution materials. We expect that such a complex response could be engineered in other polymeric materials with the requirement of two independent mechanisms leading to an opposite local change of refractive index. Further study and judicious engineering of such a novel type of highly nonlinear polymer medium may pave the way to complex patterning and novel lithographic techniques, as well as zero-epsilon nonlinear metamaterials, nonlinear optofluidics, and even nanophotonic applications due to its large attainable values of the refractive index. Notably, this induced δ*n* change is quite high in comparison to other nonlinear materials such as semiconductor crystals AlGaAs or LiNbO_3_. Recently, the self-focusing response was utilized to create low-loss deformable optical fiber interconnects.^37^ The elucidation of the light-induced material patterning mechanism can trigger new types of patterning or devices.

## Materials and Methods

The samples used in this study consisted of anionically polymerized polybutadiene (PB). The PB of 390 kg/mol was received from Polimeri Europa (Eni S.p.A.). Tetrahydrofuran (THF) was purchased from Sigma-Aldrich and was used as received. The polymer solutions were prepared under ambient conditions, at a fixed concentration of 20% polymer weight fraction (20 wt % = 0.2222 g/mL) for PB (390k).

The schematic of the experimental setup used for sample irradiation is shown in Figure 1a. Laser irradiation and imaging are happening simultaneously, under an optical microscope on the axis (*z*) perpendicular to the laser beam along the *y* direction. For this reason, a CW red laser (λ = 671 nm, various powers) was placed on a modified stage of a Zeiss Axioskop 2 optical microscope. The transmitted laser beam exiting the cell was projected on a screen. A 4× microscope objective lens (numerical aperture NA = 0.12) was used to focus the laser beam on the entrance wall of the sample cell. The beam diameter at the focal point was about 20 μm. The polymer solutions were placed in spectroscopic quartz cuvettes (Hellma, with 2, 4, and 10 mm path lengths).

The imaging of the pattern formation was achieved using a variant of the phase contrast microscopy technique. The Köhler illumination microscope was used to produce a collimated beam impinging on the polymer sample. The images were acquired by defocusing (∼100 μm above the focal plane) the microscope’s objective lens (NA = 0.15). Under such conditions, the intensity recorded on the CCD camera is related to the phase shift of the white light beam and a quantitative analysis of the refractive index difference between the light-induced pattern and the surrounding solution is allowed.^38−40^

## Supporting Information Available

Time evolution of scattering center during irradiation of THF solutions (AVI)
Bubble inflation in THF solutions triggered by laser irradiation followed by deflation when the laser is off (AVI)
Phase contrast imaging of the refractive index patterns, time-dependent split-step Fourier simulation, reconstructed refractive index profiles of the pattern in THF solutions, irreversibility of the long-time irradiation pattern, and degassing as the origin of the local decrease of refractive index (PDF)

## Supplementary Material

ph1c01917_si_001.avi

ph1c01917_si_002.avi
