# Supplementary material for: Intermixed Time-Dependent Self-Focusing and Defocusing Nonlinearities in Polymer Solutions
Source: ACS Photonics. 2022 Feb 1;9(2):722–8. doi: 10.1021/acsphotonics.1c01917 (PMC8855434; doi:10.1021/acsphotonics.1c01917)
Supplement: Supplementary file 3 — ph1c01917_si_003.pdf [file ph1c01917_si_003.pdf]

Intermixed time dependent self-focusing and defocusing nonlinearities in polymer solutions

Athanasios Bogris, [1, 2] Nikolaos A. Burger, [1, 2] Konstantinos G. Makris, [1,3] Benoit Loppinet, [1*] George Fytas, [1,4*]

[1] FORTH, Institute of Electronic Structure and Laser, 70013 Heraklion, Crete, Greece

[2] Department of Materials Science and Technology, University of Crete, 70013 Heraklion, Crete, Greece

[3] Department of Physics, University of Crete, Heraklion 71003, Greece

[4] Max-Planck Institute for Polymer Research, 55128 Mainz, Germany

Correspondence: benoit@iesl.forth.gr, fytas@mpip-mainz.mpg.de

SUPPLEMENTARY INFORMATION:

Phase contrast imaging of the refractive index patterns

Time dependent split step Fourier simulation

Reconstructed refractive index profile of pattern in THF solutions

Irreversibility of the long time irradiation pattern

Degassing as the origin of the local decrease of refractive index

Phase contrast imaging of the refractive index patterns:

We used a variant of phase contrast imaging, well adapted to the cylindrical symmetry of the irradiation and patterning. [40] The refractive index pattern is imaged using a transmission optical microscope with a collimated beam illumination and a slightly defocused objective (x5) lens. Under those conditions and for cylindrically symmetrical patterns, the formed image relates to the radial refractive index profile at

different position y along the laser propagation axis, $n(r, y)$ through the following relation:

$$\frac{I(x,y,t)}{I_0} = k_0^2 \Delta z \frac{\partial^2}{\partial x^2} \int_x^\infty \frac{n(r,y) r dr}{\sqrt{r^2 - x^2}} \qquad \text{(S1)}$$

where I_0 is the in focus image, $I(x, y, t)$ is the imaged recorded at defocus Δz, and k_0 is the wave vector of the light used for imaging, x denotes the axis perpendicular to the laser propagation axis y and to the imaging axis z. [39,41]

We used as a simple measure of the kinetic evolution, the image contrast defined as $I^*(y, t) = \frac{I(x=0,y,t)}{I(x=0,y,t=0)}$ where $x = 0,\ y = 0$ is the center of the beam at the cell entrance. The I^* reported from experiments have been averaged over several pixels along the propagation axis y to obtain better statistics.

If the refractive index profile has a Gaussian like profile $n(r) = A\, exp(-\frac{r^2}{a^2})$ then $I^*(y, t) \sim \frac{A(y,t)}{a(y,t)}$, reflecting the gradient of refractive index. For more complex profiles, the relation of I^* with $n(r)$ can become intricate. In the case of mixtures where n(r) is well described by a sum of two Gaussian, then $I^*(y, t) \sim \frac{A_+(y,t)}{a_+(y,t)} - \frac{A_-(y,t)}{a_-(y,t\,)}$, where A+ and A- are the amplitude of the positive and negative refractive index profiles, and a+ and a- are the corresponding width of the patterns, Experimentally $a_+ \sim 10\ \mu m$ and $a_- \sim 20\ \mu m$.

Ideally one would transform the experimental images into refractive index map. However, the images are too noisy for this approach. We have then preferred to produce phase images from the simulated refractive index map to allow a good comparison between the simulation results and the experimental results. The phase contrast image of the simulation $n(r)$ was computed according to Equation S1.

To do so, we make use of the relation linking the inverse and direct Abel transform AT^{-1} and AT:

$$\frac{\partial^2}{\partial x^2} AT\big(n(r)\big) = -\pi \frac{\partial}{\partial x}\Big(x\, AT^{-1}\big(n(r)\big)\Big) \qquad \text{(S2)}$$

Equation S2 was implemented in the Matlab code, using an inverse Abel transform routine. [42]

Time dependent split step Fourier simulation:

Equation 1 in the main text governs the propagation of light in a nonlinear medium under the paraxial approximation for the case of self-focusing and/or defocusing nonlocal nonlinearity in a photoreactive polymer medium. [30] Here we implemented a time dependent symmetrized split step Fourier (SSF) method. In its normalized form the propagation equation is

$$i\frac{\partial\phi}{\partial y}+\frac{1}{2k_0 n}\frac{\partial^2\phi}{\partial x^2}+V(x,y,t)\phi=0$$

The numerical solution was obtained on a spatial and time grid, by using discrete Fourier transforms (DFT). The DFT of a vector is computed by a fast Fourier transform (FFT) algorithm or its inverse (IFFT). The three steps of the final algorithm for fixed optical potential are:

$$\phi_1(n\Delta x, y_0+\Delta y/2)=\ IFFT\{FFT[\phi(n\Delta x, y_0)]\exp(-ik^2\Delta y/2)\},$$

$$\phi_2(n\Delta x, y_0+\Delta y/2)=\exp(i\Delta y V(n\Delta x))\phi_1(n\Delta x, y_0+\Delta y/2),$$

$$\phi_3(n\Delta x, y_0+\Delta y/2)=\ IFFT\{FFT[\phi_2(n\Delta x, y_0+\Delta y/2)]\exp(-ik^2\Delta y/2)\},$$

where k is a discretized wave number.

. We highlight that the SSF method was adopted in our approach, as it performs faster calculations with greater accuracy. For our non-local in time problem we apply the SSF algorithm for every step-in time. First, we discretize the time. Then given an initial condition for the field we calculate the diffraction pattern and store the resulted pattern (refractive index map). Such pattern provides us with the potential (z-dependent) for the next step in time. In every step we add everything from the previous steps. After many steps in time we arrive at the desired result. The integral of Equation (2) or Equation (3) is calculated numerically by an approximate Riemann sum or by applying the trapezoidal rule. Comparison between simulation and experiments required the following scaling factors between the normalized coordinates $\tilde{x}, \tilde{y}$, and the

physical-experimental ones: $x = \tilde{x}\ x_0$, $z = \tilde{z}\ /(2k_0 n_0 x_0^2)$, with $x_0 = \frac{1}{k_0}\sqrt{\frac{1}{2n_0\Delta}}$, where Δ relates to the maximum of the refractive index modulation as $V_s = \delta n_s / \Delta$. Time is left in arbitrary units, and match to the experimental results through the comparison with experimental kinetics. The simulation was implemented in Matlab. No attempt was made to obtain quantitative comparison, but merely to obtain qualitative agreement between simulations and experiments.

<u>Reconstructed refractive index profile of pattern in THF solutions:</u>

Reconstruction of the refractive index profile at the entrance of the beam is shown in Figure S1. A double integration was used and a reverse Abel transform.

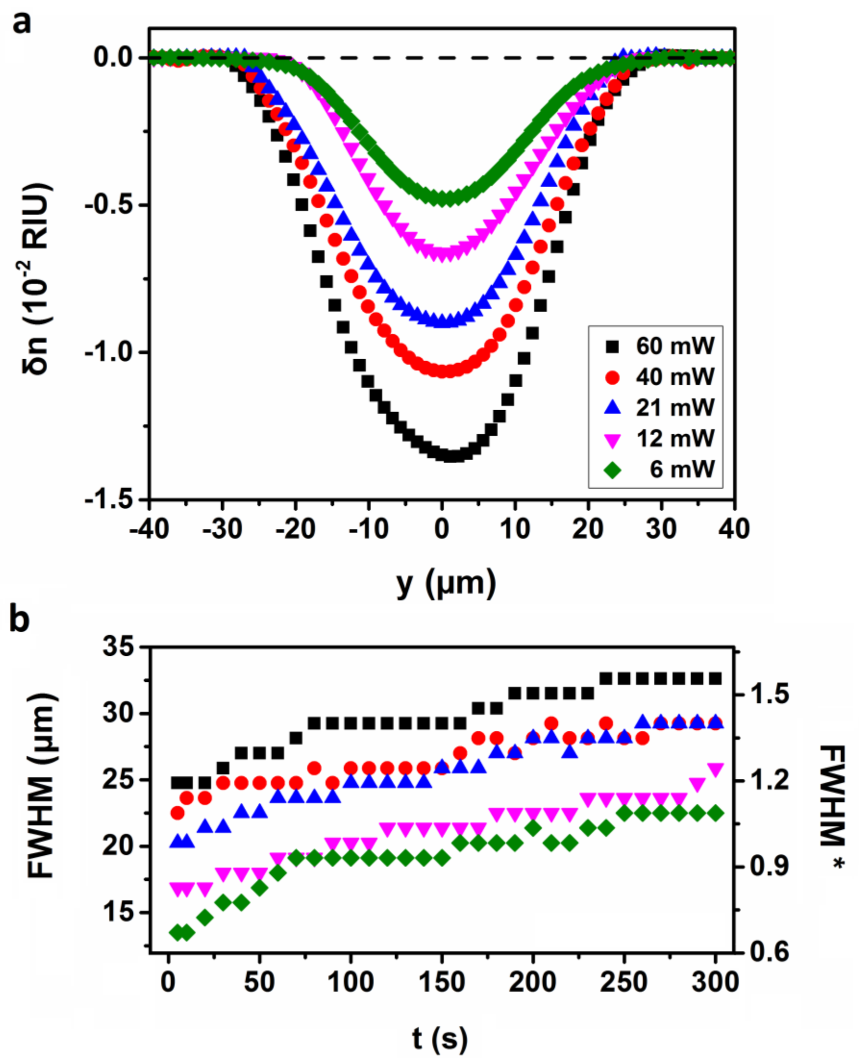

Figure S1: a. Refractive index profiles for the written patterns in PB (390k) 20wt% solution in THF after 300s of illumination. The colored symbols refer to different laser power used as indicated in the plot. b. Time evolution of full width half maximum

(FWHM) of the light induced patterns close to the focal point (entrance wall of the sample cell), for PB (390k) solutions in THF (c=20wt %) at different laser powers (P). FWHM* refers to the resized full width half maximum of the induced patterns, resized by the beam waist.

Based on the reconstruction, the refractive index profile assumes a near Gaussian shape. The pattern's full width at half maximum close to the beam entrance exhibited a slight increase with irradiation time before reaching a steady state value (Figure S1b).

Note that in phase contrast microscopy I* depends on the refractive index gradient $I^{*}\sim\frac{\delta n}{R}$ or $\delta n\sim I^{*}R$ where R is the radius of the pattern. We have neglected the change of R in our simplified analysis as the small variation is not expected to have consequences on the results.

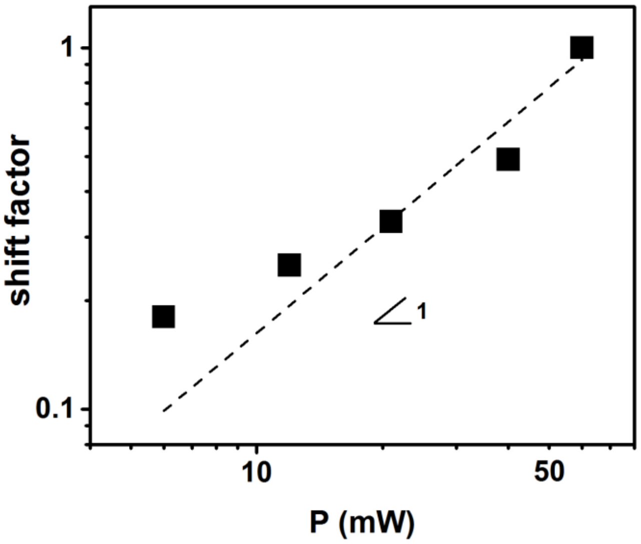

Figure S2: Shift factor used for the superimposition of the experimental averaged normalized intensity (I*) curves for PB (390k) 20wt % solution in THF as a function of laser power (P).

Table S1: Parameters of the numerical simulations for producing results of Figures 5 & 6.

N_t	t_0	S	z_{max}	u_{in0}	w	V_{s+}	V_{s-}	V_{a+}	V_{a-}	V_c
150	150	1000	50	200	15	0.25	-0.32	600	2000	20

<u>Irreversibility of the long-time irradiation pattern</u>

Prolonged irradiation of PB solutions in THF led to the formation of irreversible and stable light-induced structures, impossible to be dissolved in a pure solvent. We can conjecture that the specific response of THF solutions is linked to THF itself, an organic compound which is not very stable as it easily forms peroxides.[43] Then crosslinking through a free radical generation mechanism could be a possible scenario, but it should be of a different nature comparing to the "positive" case, where refractive index changes depend on concentration. Therefore, THF molecules, free radicals and peroxides may be integral part of the process. Chemical alterations of the polymer microstructure due to laser irradiation could lead to a change in the dn/dc. Spectroscopic measurements on the extracted structure after irradiation could potentially give answers on this, but this is the subject of a future work, as here we focus on the optical response of such systems.

Degassing as the origin of the local decrease of refractive index

One possible explanation for the local decrease of refractive index, would be the occurrence of gas products that would result from a photochemical process. That would form small bubbles and reduce the overall refractive index. [34] This implies a very slow diffusion of the formed nanobubbles in the material. Assuming a simple effective medium approximation for the refractive index, a few % of gas inclusion would lead to the 10^{-2} RIU decrease. The gas emission scenario is supported by the observation of occasional existence of "large" gas inclusion (bubbles) in semidilute polybutadiene solutions in THF (20wt %). When the laser beam hit the bubbles (probably formed during solution preparation), they immediately expand their volume, almost 50% comparing to the initial one before irradiation, as can be seen in Figure S3. The bubbles deflated when light was turned off. It is worth noticing that no such expanding bubbles were observed in irradiated PB solutions in alkane or other solvents. A video of similar (but smaller) laser sensitive bubbles is available in SI ACS website (PI_THF_bubbles(compressed).avi). The view is about 100 μm x 100μm. A created bubble deflates when the laser light is removed, and another bubble forms when the laser light is brought back (30s in the video) .

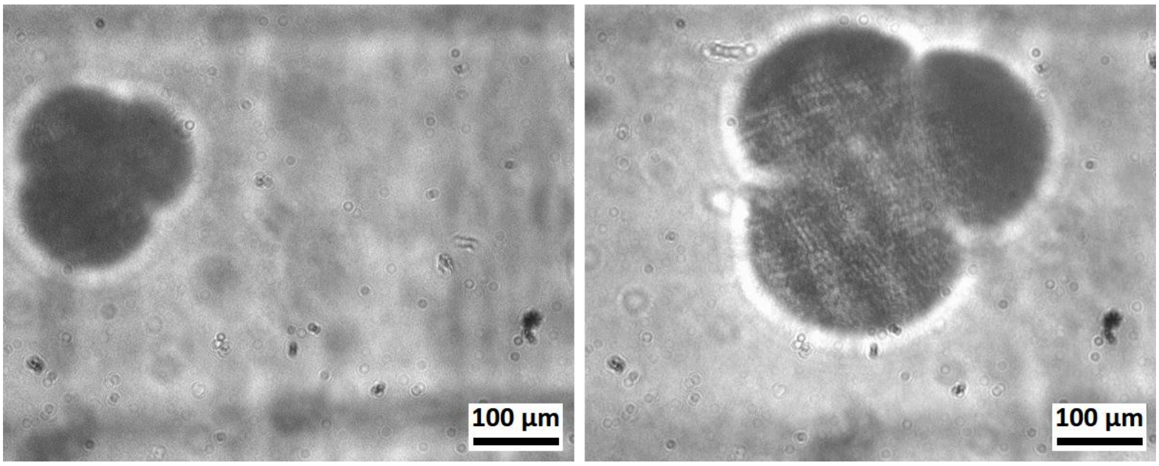

Figure S3: Response of gas inclusion present in semidilute PB (390k) solution in THF (20wt %) to laser irradiation. Left: Gas inclusion present away from laser light. Right: Expansion of gas inclusion when hit by the red laser beam. Laser power: 60mW. A video sequence of similar but smaller bubble inflation deflation during irradiation in THF solution is available in SI materials. A pre-formed bubble (bright spot due to the laser scattering) is seen to deflate and then after some small sample position readjustment another bubble reforms and deflates when laser is removed. Video of similar effect is available in PI_THF_Bubbles_compressed.avi.

Another observation in favor of gas emission in THF solution under irradiation is the presence of bubbles in more dilute solutions under red light irradiation. A polyisoprene (1090k) solution in THF (1wt % / 2.5 c*) under irradiation was observed under microscope. Many scattering centers observed sparkling, randomly placed in the scattering volume (Figure S4). No such “sparkling” were observed in hexane solutions under similar conditions.

The incorporation of gas in THF solutions would certainly decrease the refractive index, which is consistent with the experimental observations.

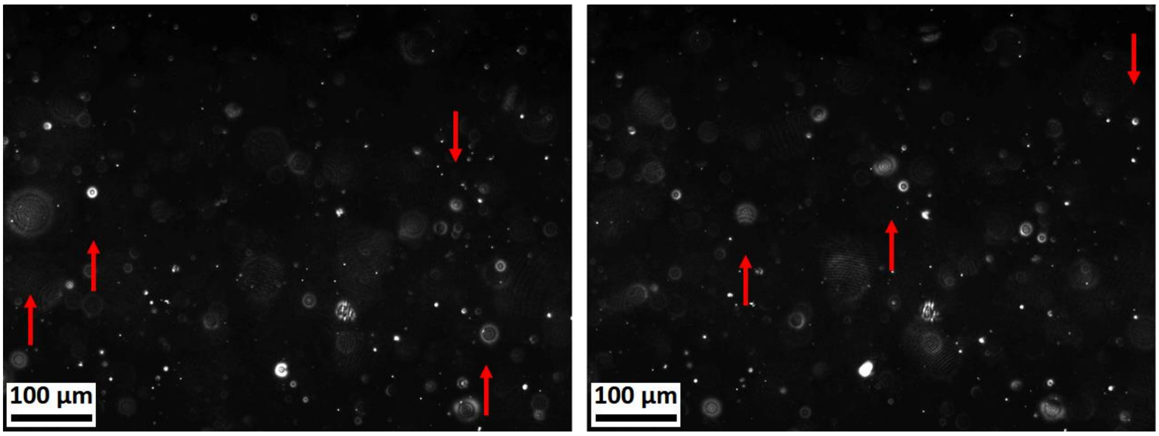

Figure S4: Scattering centers in PI (1090k) solutions in THF (1wt %) under irradiation. The whole field of view of the image is inside the scattering volume. The two different

images are different illumination moments (difference of seconds) and red arrows are pointing out scattering centers that do not show in the other image.

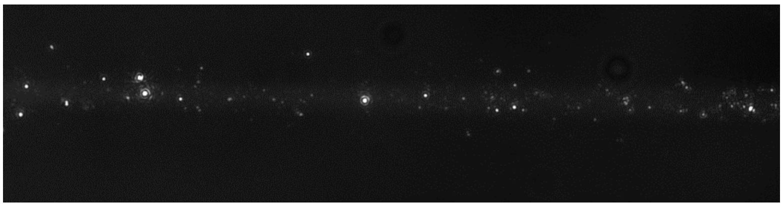

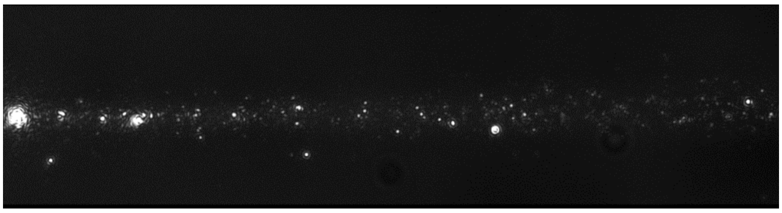

Figure S5 : Scattering images during the irradiation (50mW) of 15% PI in THF (after few sec (up) and after 6 min (down)). Not shown here is that the beam opens during the process (defocusing case) and that a lower refractive index pattern becomes visible through phase contrast microscopy. The point scatterers appear to increase in number and also more centered as irradiation goes on. Note that in the phase image pictures the scatterers are invisible, sign of small size (below micron). It is worth mentioning that though clearly imaged the scattering is not very strong and does not affect the overall transmission. We tentatively attribute them to nanobubbles which are too small to be resolve by phase contrast microscopy, but with large enough scattering cross section to be seen in the scattering images. Height of the picture is about 100 µm. The full sequence is available as video in false color in SI materials (PI_THF_scattering.avi). The effect is best seen in accelerated mode (for example using the slide bar in video viewer)

References

1. Menzel, R., *Photonics: Linear and Nonlinear Interactions of Laser Light and Matter*. **2001**.

2. Khoo, I. C., Nonlinear Optics of Liquid Crystalline Materials. *Physics Reports* **2009,** *471* (5), 221-267.

3. Voit, A.; Krekhov, A.; Enge, W.; Kramer, L.; Köhler, W., Thermal Patterning of a Critical Polymer Blend. *Physical Review Letters* **2005,** *94* (21), 214501.

4. Merino, E.; Ribagorda, M., Control over Molecular Motion Using the Cis-Trans Photoisomerization of the Azo Group. *Beilstein journal of organic chemistry* **2012,** *8*, 1071-1090.

5. Villafranca, A. B.; Saravanamuttu, K., An Experimental Study of the Dynamics and Temporal Evolution of Self-Trapped Laser Beams in a Photopolymerizable Organosiloxane. *The Journal of Physical Chemistry C* **2008,** *112* (44), 17388-17396.

6. Ambrosio, A.; Maddalena, P.; Marrucci, L., *Molecular Model for Light-Driven Spiral Mass Transport in Azopolymer Films*. **2013**; Vol. 110.

7. Voit, A.; Krekhov, A.; Köhler, W., Laser-Induced Structures in a Polymer Blend in the Vicinity of the Phase Boundary. *Physical Review E* **2007,** *76* (1), 011808.

8. Królikowski, W.; Bang, O.; Rasmussen, J.; Wyller, J., Modulational Instability in Nonlocal Nonlinear Kerr Media. *Physical review. E, Statistical, nonlinear, and soft matter physics* **2001,** *64*, 016612.

9. Conti, C.; Peccianti, M.; Assanto, G., Route to Nonlocality and Observation of Accessible Solitons. *Physical review letters* **2003,** *91*, 073901.

10. Conti, C.; Peccianti, M.; Assanto, G., Observation of Optical Spatial Solitons in a Highly Nonlocal Medium. *Physical review letters* **2004,** *92*, 113902.

11. Peccianti, M.; Conti, C.; Assanto, G.; De Luca, A.; Umeton, C., Routing of Anisotropic Spatial Solitons and Modulational Instability in Liquid Crystals. *Nature* **2004,** *432* (7018), 733-737.

12. Makris, K. G.; Sarkissian, H.; Christodoulides, D. N.; Assanto, G., Nonlocal Incoherent Spatial Solitons in Liquid Crystals. *J. Opt. Soc. Am. B* **2005,** *22* (7), 1371-1377.

13. Rotschild, C.; Cohen, O.; Manela, O.; Segev, M.; Carmon, T. In *Solitons in Nonlinear Media with Infinite Range of Nonlocality: First Observation of Coherent Elliptic Solitons and Bright Vortex-Ring Solitons*, Nonlinear Guided Waves and Their Applications, Dresden, 2005/09/06; Optical Society of America: Dresden, 2005; p FC2.

14. Rotschild, C.; Alfassi, B.; Cohen, O.; Segev, M., Long-Range Interactions between Optical Solitons. *Nature Physics* **2006,** *2* (11), 769-774.

15. Rotschild, C.; Segev, M.; Xu, Z.; Kartashov, Y.; Torner, L.; Cohen, O., Two-Dimensional Multipole Solitons in Nonlocal Nonlinear Media. *Optics letters* **2006,** *31*, 3312-4.

16. Alfassi, B.; Rotschild, C.; Manela, O.; Segev, M.; Christodoulides, D., Nonlocal Surface-Wave Solitons. *Physical review letters* **2007,** *98*, 213901.

17. Alfassi, B.; Rotschild, C.; Manela, O.; Segev, M.; Christodoulides, D. N., Boundary Force Effects Exerted on Solitons in Highly Nonlocal Nonlinear Media. *Optics Letters* **2007,** *32* (2), 154-156.

18. Kaminer, I.; Rotschild, C.; Manela, O.; Segev, M., Periodic Solitons in Nonlocal Nonlinear Media. *Optics Letters* **2007,** *32* (21), 3209-3211.

19. Efremidis, N., Nonlocal Lattice Solitons in Thermal Media. *Physical Review A* **2008,** *77*, 063824.

20. Towers, I.; Malomed, B. A., Stable (2+1)-Dimensional Solitons in a Layered Medium with Sign-Alternating Kerr Nonlinearity. *J. Opt. Soc. Am. B* **2002,** *19* (3), 537-543.

21. Pelinovsky, D.; Kevrekidis, P.; Frantzeskakis, D., Averaging for Solitons with Nonlinearity Management. *Physical review letters* **2004,** *91*, 240201.

22. Centurion, M.; Porter, M.; Kevrekidis, P.; Psaltis, D., Nonlinearity Management in Optics: Experiment, Theory, and Simulation. *Physical review letters* **2006,** *97*, 033903.

23. Smith, V.; Leung, B.; Cala, P.; Chen, Z.; Man, W., Giant Tunable Self-Defocusing Nonlinearity and Dark Soliton Attraction Observed in M-Cresol/Nylon Thermal Solutions. *Opt. Mater. Express* **2014,** *4* (9), 1807-1812.

24. Sigel, R.; Fytas, G.; Vainos, N.; Pispas, S.; Hadjichristidis, N., Pattern Formation in Homogeneous Polymer Solutions Induced by a Continuous-Wave Visible Laser. *Science (New York, N.Y.)* **2002,** *297*, 67-70.

25. Anyfantakis, M.; Loppinet, B.; Fytas, G.; Pispas, S., Optical Spatial Solitons and Modulation Instabilities in Transparent Entangled Polymer Solutions. *Opt. Lett.* **2008,** *33*, 2839-2841.

26. Loppinet, B.; Somma, E.; Vainos, N.; Fytas, G., Reversible Holographic Grating Formation in Polymer Solutions. *J. Am. Chem. Soc.* **2005,** *127*, 9678-9679.

27. Anyfantakis, M.; Königer, A.; Pispas, S.; Köhler, W.; Butt, H.-J.; Loppinet, B.; Fytas, G., Versatile Light Actuated Matter Manipulation in Transparent Non-Dilute Polymer Solutions. *Soft Matter* **2012,** *8*, 2382-2384.

28. Kewitsch, A. S.; Yariv, A., Self-Focusing and Self-Trapping of Optical Beams Upon Photopolymerization. *Optics Letters* **1996,** *21* (1), 24-26.

29. Shoji, S.; Kawata, S.; Sukhorukov, A. A.; Kivshar, Y. S., Self-Written Waveguides in Photopolymerizable Resins. *Optics Letters* **2002,** *27* (3), 185-187.

30. Biria, S.; Morim, D. R.; An Tsao, F.; Saravanamuttu, K.; Hosein, I. D., Coupling Nonlinear Optical Waves to Photoreactive and Phase-Separating Soft Matter: Current Status and Perspectives. *Chaos* **2017,** *27*, 104611.

31. Fleck, J. A.; Morris, J. R.; Feit, M. D., Time-Dependent Propagation of High Energy Laser Beams through the Atmosphere. *Applied physics* **1976,** *10* (2), 129-160.

32. Okamoto, K., *Fundamentals of Optical Waveguides*. Elsevier Science: **2006**.

33. Askadskii, A. A., Influence of Crosslinking Density on the Properties of Polymer Networks. *Polymer Science U.S.S.R.* **1990,** *32* (10), 2061-2069.

34. D'Amico, C.; Caillaud, C.; Velpula, P. K.; Bhuyan, M. K.; Somayaji, M.; Colombier, J. P.; Troles, J.; Calvez, L.; Nazabal, V.; Boukenter, A.; Stoian, R., Ultrafast Laser-Induced Refractive

Index Changes in Ge15as15s70 Chalcogenide Glass. *Opt. Mater. Express* **2016,** *6* (6), 1914-1928.

35. Anyfantakis, M.; Fytas, G.; Mantzaridis, C.; Pispas, S.; Butt, H.-J.; Loppinet, B., *Experimental Investigation of Long Time Irradiation in Polydiene Solutions: Reversibility and Instabilities*. **2010**; Vol. 12.

36. Anyfantakis, M.; Pamvouxoglou, A.; Mantzaridis, C.; Pispas, S.; Butt, H. J.; Fytas, G.; Loppinet, B., Kinetics of Light-Induced Concentration Patterns in Transparent Polymer Solutions. *J. Phys. Chem. B* **2017,** *121*, 7180-7189.

37. Barty, A.; Nugent, K.; Paganin, D.; Roberts, A., Quantitative Optical Phase Microscopy. *Opt. Lett.* **1998,** *23*, 817-819.

38. Barone-Nugent, E.; Barty, A.; Nugent, K., Quantitative Phase-Amplitude Microscopy I: Optical Microscopy. *J. Microsc.* **2002,** *206*, 194-203.

39. Roberts, A.; Ampem-Lassen, E.; Barty, A.; Nugent, K. A.; Baxter, G. W.; Dragomir, N.; Huntington, S., Refractive-Index Profiling of Optical Fibers with Axial Symmetry by Use of Quantitative Phase Microscopy. *Opt. Lett.* **2002,** *27*, 2061-2063.

40. Anyfantakis, M.; Fytas, G.; Mantzaridis, C.; Pispas, S.; Butt, H. J.; Loppinet, B., Experimental Investigation of Long Time Irradiation in Polydiene Solutions: Reversibility and Instabilities. *Journal of Optics, v.12 (2010)* **2010,** *12* (12), 124013.

41. Ampem-Lassen, E.; Huntington, S. T.; Dragomir, N. M.; Nugent, K. A.; Roberts, A., Refractive Index Profiling of Axially Symmetric Optical Fibers: A New Technique. *Opt. Express* **2005,** *13* (9), 3277-3282.

42. Kasim, M. F.; Holloway, J.; Ceurvorst, L.; Levy, M. C.; Ratan, N.; Sadler, J.; Bingham, R.; Burrows, P. N.; Trines, R.; Wing, M.; Norreys, P., Quantitative Single Shot and Spatially Resolved Plasma Wakefield Diagnostics. *Physical Review Special Topics - Accelerators and Beams* **2015,** *18* (8), 081302.

43. Müller, H., Tetrahydrofuran. In *Ullmann's Encyclopedia of Industrial Chemistry*, **2000**.
